# Candidate Genes Modulating Reproductive Timing in Elite US Soybean Lines Identified in Soybean Alleles of *Arabidopsis* Flowering Orthologs With Divergent Latitude Distribution

**DOI:** 10.3389/fpls.2022.889066

**Published:** 2022-04-29

**Authors:** Nicholas Dietz, Yen On Chan, Andrew Scaboo, George Graef, David Hyten, Mary Happ, Brian Diers, Aaron Lorenz, Dechun Wang, Trupti Joshi, Kristin Bilyeu

**Affiliations:** ^1^Division of Plant Science and Technology, University of Missouri, Columbia, MO, United States; ^2^Christopher S. Bond Life Sciences Center, University of Missouri, Columbia, MO, United States; ^3^MU Data Science and Informatics Institute, University of Missouri, Columbia, MO, United States; ^4^Department of Agronomy and Horticulture, University of Nebraska, Lincoln, NE, United States; ^5^Department of Crop Sciences, University of Illinois, Urbana, IL, United States; ^6^Department of Agronomy and Plant Genetics, University of Minnesota, Saint Paul, MN, United States; ^7^Department of Plant, Soil and Microbial Sciences, Michigan State University, East Lansing, MI, United States; ^8^Department of Electrical Engineering and Computer Science, University of Missouri, Columbia, MO, United States; ^9^Department of Health Management and Informatics, School of Medicine, University of Missouri, Columbia, MO, United States; ^10^USDA/ARS Plant Genetics Research Unit, Columbia, MO, United States

**Keywords:** development, soybean, flowering time, vegetative phase, reproductive phase, genomics, orthologs

## Abstract

Adaptation of soybean cultivars to the photoperiod in which they are grown is critical for optimizing plant yield. However, despite its importance, only the major loci conferring variation in flowering time and maturity of US soybean have been isolated. By contrast, over 200 genes contributing to floral induction in the model organism *Arabidopsis thaliana* have been described. In this work, putative alleles of a library of soybean orthologs of these *Arabidopsis* flowering genes were tested for their latitudinal distribution among elite US soybean lines developed in the United States. Furthermore, variants comprising the alleles of genes with significant differences in latitudinal distribution were assessed for amino acid conservation across disparate genera to infer their impact on gene function. From these efforts, several candidate genes from various biological pathways were identified that are likely being exploited toward adaptation of US soybean to various maturity groups.

## Introduction

**FIGURE 1:**
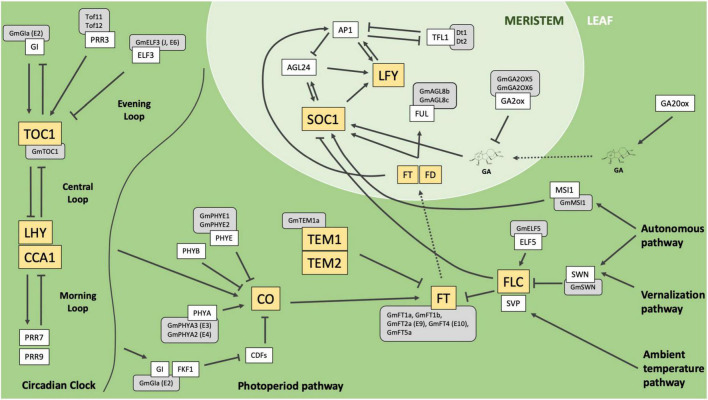
Simplified depiction of characterized pathways and genes which influence flowering in the model organism *Arabidopsis thaliana* (adapted from Fornara et al., 2010). Sharp boxes represent *Arabidopsis* genes. Gray rounded boxes represent orthologous genes in soybean. Yellow boxes highlight key integrators or regulators in their respective pathways. Solid arrows indicate positive regulation, T-bars represent negative regulation, dotted arrows represent mobility from leaf to meristem. *Arabidopsis* genes: *Gigantea* (*GI*), *Pseudo Response Regulator 3* (*PRR3*), *Early Flowering 3 (ELF3), Timing of CAB Expression 1 (TOC1), Late Elongated Hypocotyl (LHY), Circadian Clock Associated 1 (CCA1), Pseudo Response Regulator 7 (PRR7), Pseudo Response Regulator 9 (PRR9), Flavin-binding, Kelch Repeat, F-Box 1 (FKF1), Cycling DOF Factors (CDFs), Phytochrome A (PHYA), Phytochrome B (PHYB), Phytochrome E (PHYE), Constans (CO), Tempranillo 1 (TEM1), Tempranillo 2 (TEM2), Flowering Locus T (FT), Flowering Locus C (FLC), Early Flowering 5 (ELF5), Short Vegetative Phase (SVP), Swinger (SWN), Multicopy Suppressor of IRA 1 (MSI1), Gibberellin 20-oxidase (GA20ox), Gibberellin 2-oxidase (GA2ox), Fruitful (FUL), Flowering Locus D (FD), Suppressor of Constans 1 (SOC1), Leafy (LFY), Agamous-like 24 (AGL24), Apetala 1 (AP1), Terminal Flower 1 (TFL1).* Soybean genes: *Gigantea A (GmGIa, E2), Time of Flowering 11 (Tof11), Time of Flowering 12 (Tof12), Early Flowering 3 (GmELF3, J, E6), Timing of CAB Expression 1 (GmTOC1), Phytochrome A3 (GmPHYA3, E3), Phytochrome A2 (GmPHYA2, E4), Phytochrome E1 (PHYE1), Phytochrome E2 (PHYE2), Tempranillo 1a (GmTEM1a), Flowering Locus T 1a (GmFT1a), Flowering Locus T 1b (GmFT1b), Flowering Locus T 2a (GmFT2a, E9), Flowering Locus T 4 (GmFT4, E10), Flowering Locus T 5a (GmFT5a), Early Flowering 5 (GmELF5), Swinger (GmSWN), Multicopy Suppressor of IRA1 (GmMSI1), Gibberellins 2-oxidase 5 (GmGA2OX5), Gibberellins 2-oxidase 6 (GmGA2OX6), Agamous-like 8b (GmAGL8b), Agamous-like 8c (GmAGL8c), Determinant 1 (Dt1), Determinant 2 (Dt2).*

Flowering time is a key trait for maximizing yield potential in many crop species. Extensive research into the genetic mechanisms controlling reproductive timing in the long-day-flowering model organism *Arabidopsis thaliana* has blazed the trail for understanding the flowering process and has been instrumental in identifying these genes in other species. At least six major pathways coordinate to modulate flowering, including the photoperiod, ambient temperature, vernalization, autonomous, aging, and gibberellins pathways (reviewed in Fornara et al., 2010). Together, members of these pathways integrate environmental and endogenous signals and converge upon key floral integrator genes to promote flowering under optimum conditions (Figure 1).

The circadian clock complex is composed of three distinct groups of genes, the morning loop, central loop, and evening loop, which regulate one another to maintain an endogenous 24-h oscillation period. *LATE ELONGATED HYPOCOTYL* (*LHY*) and *CIRCADIAN CLOCK-ASSOCIATED 1* (*CCA1*) promote expression of members of the morning loop, while *TIMING OF CAB EXPRESSION 1* (*TOC1*) regulates members of the evening loop (Alabadì et al., 2001; Gendron et al., 2012). Reciprocal regulation between these three loops ensures proper expression of day/night developmental programs. Photoreceptors in the photoperiod pathway, such as the phytochrome and cryptochrome molecules, perceive different wavelengths of light and, together with output from the circadian clock, affect stabilization of the *CONSTANS* (*CO*) protein in the leaf (Valverde et al., 2004). The relative balance of *CO*, a floral promoter, and the *TEMPRANILLO* (*TEM1*, *TEM2*) repressor genes regulates expression of *FLOWERING LOCUS T* (*FT*), a key florigen producing gene, in response to daylength (Castillejo and Pelaz, 2008). *FLOWERING LOCUS C* (*FLC*) integrates signaling from both the vernalization and autonomous pathways and, when expressed, functions to delay flowering by repressing key floral integrators *SUPPRESSOR OF OVEREXPRESSION OF CONSTANS 1* (*SOC1*) and *FT* expression (Lee et al., 2000).

Under optimum flowering conditions, *FT* is expressed in the leaf and produces a mobile florigen that travels to the meristem by way of the phloem. Once in the meristem, *FT*, in concert with *SOC1* and *LEAFY* (*LFY*), stimulate the floral meristem transition and activate homeotic genes that give rise to the various floral organs (Parcy et al., 1998; Liu C. et al., 2009). Like *FT*, gibberellins, a class of hormone involved in broad developmental regulation throughout the plant, are produced in the leaf and travel to the meristem where they promote floral induction by upregulation of *SOC1* (Mutasa-Göttgens and Hedden, 2009).

In contrast to *Arabidopsis*, soybean begins its reproductive period in response to short day photoperiods and lacks a vernalization requirement for proper seed germination. Despite this evolutionary divergence, nearly all the isolated flowering time genes in soybean are orthologs of *Arabidopsis* genes. *GmFT2a* (*E9*) and *GmFT5a* are two such orthologs of the *Arabidopsis FT* gene which promote flowering in response to environmental and endogenous signals under appropriate conditions (Takeshima et al., 2016; Zhao et al., 2016). *E1*, a legume-specific B3-related transcription factor, mediates the photoperiod response to suppress *GmFT2a* and *GmFT5a* expression during long days (Xia et al., 2012). Once days shorten past a critical threshold, *E1* is suppressed, which derepresses *GmFT2a* and *GmFT5a* to allow flowering.

*Tof11* and *Tof12* are recently isolated paralogs which contributed to soybean domestication (Lu et al., 2020). These genes are orthologs of the *Arabidopsis* circadian clock gene *PSEUDO RESPONSE REGULATOR 3* (*PRR3*), which function upstream of the central loop oscillator *GmLHY*. *GmPHYA3* (*E3*) and *GmPHYA2* (*E4*) are orthologs of the *Arabidopsis PHYA* gene (Liu et al., 2008; Watanabe et al., 2009). These phytochrome molecules perceive red and far-red light, which, in concert with the *Tof11*, *Tof12*, and *GmLHY*, mediate *E1* expression to coordinate flowering in response to daylength. Like *Tof11* and *Tof12*, *GmGIa* (*E2*) is a circadian clock-associated member of the evening loop in soybean and is a paralog of the *Arabidopsis GIGANTEA* (*GI*) gene (Watanabe et al., 2011). *GmGIa* contributes to flowering in response to photoperiod by delaying expression of *GmFT2a* under long days. The *J* locus (*E6*) is an ortholog of *EARLY FLOWERING 3* (*ELF3*), another circadian clock gene which delays flowering under short day photoperiods and is used to extend the vegetative phase of soybean grown in low latitude production environments (Lu et al., 2017).

Five mutant alleles of the *E1* gene have been identified, including alleles containing a frameshift mutation (*e1-fs*), a missense mutation (*e1-as*), a retrotransposon insertion (*e1-re*), a likely regulatory mutation (*e1-p*), and a complete deletion of the *E1* gene (*e1-nl*) (Tsubokura et al., 2013). A recessive allele of *E2* contains a single base substitution resulting in a non-sense mutation (Watanabe et al., 2011). The *E3* gene has two variant alleles that differ in their amino acid sequences, relative to the Williams 82 reference: a rare allele containing a non-sense mutation in third exon (*e3-mo*), and an allele containing a large ∼13 kb deletion after the third exon, resulting in a truncated protein (*e3-tr*) (Watanabe et al., 2009; Tsubokura et al., 2013). A single mutant allele of the *E4* gene contains retrotransposon insertion in its first exon (*e4-SORE-1*) (Liu et al., 2008).

Soybean varieties are adapted to narrow latitudinal ranges, referred to as Maturity Group (MG), due to their propensity to flower and mature in response to photoperiod. In US soybean production, *E1, E2, and E3* confer most of the observed variation in flowering time and maturity; no variation in the *E4* gene has been detected in US soybean cultivars (Langewisch et al., 2017). In each case, the dominant allele confers later flowering and maturity. *e1-as*, a partially functional recessive allele of *E1*, contains a single missense mutation in its nuclear localization sequence, impairing its ability to enter the nucleus to suppress *GmFT2a*, and resulting in earlier flowering (Xia et al., 2012). The non-sense mutation in the *E2* gene results in a non-functional protein and confers early flowering (Watanabe et al., 2011). The predominant recessive allele of *E3* among US soybean, *e3-tr*, produces a truncated non-functional protein which confers early flowering (Watanabe et al., 2009). However, given the inability of short-read sequencing data to capture large insertions and deletions, the *e3-tr* mutant allele was not incorportated in this study.

Allelic combinations of these three genes are the major loci conferring adaptation to the various MGs in US soybean varieties (Langewisch et al., 2017). The dominant alleles of all three genes are present in the late flowering cultivars of the south (MG V through MG VIII), whereas accruement of additional recessive alleles confers earlier flowering of cultivars in northern MGs (MG 0–MG IV). Despite this knowledge, our understanding of the loci which confer modest changes in flowering time, such as those within MGs or between adjacent maturity groups, is still lacking. In this work, we utilized resequencing data from 264 elite US soybean lines, converted into a specialized “allele” format, to test soybean orthologs of *Arabidopsis* flowering genes for differences in latitudinal adaptation between lines differing for alleles. Here, latitude was utilized as a proxy for relative flowering time. For genes with significant *p* values, weblogos were generated from multiple sequence alignments to determine which mutations occurred in conserved peptide domains and were thus likely to impact protein function. Based on these criteria, we present a set of eight high confidence genes which warrant further investigation as targets for optimizing flowering time and maturity in US soybean cultivars.

## Materials and Methods

### Curation of Imputed Resequencing Dataset

As part of a parallel effort to catalog the extent of genetic variation in wild and cultivated soybean, our research group previously developed a diversity panel derived from two sets of publicly available resequenced soybean accessions (Zhou et al., 2015; Valliyodan et al., 2021). In this work we used 772 accessions from this diversity panel as a core set of accessions, from which we obtained a total of ∼35.7 M SNP and InDel positions (Škrabišová et al., 2022). We genotyped an additional 518 resequenced accessions at those ∼35.7 Mil positions from other published datasets (Happ et al., 2019; Liu et al., 2020) or from our own skim resequencing efforts (Supplementary Table 1). The set of 65 accessions resequenced by our research group were predominantly elite US soybean lines (ELs) derived from the public university soybean breeding programs in Minnesota, Michigan, Illinois, and Missouri.

Raw reads for publicly available accessions were obtained from NCBI SRA (projects: SRP062245, SRP105183, SRP045129, PRJNA512147) or from the National Genomics Data Center Genome Sequence Archive (project: PRJCA002030). All reads were aligned to the Wm82.a2.v1 Phytozome reference genome using BWA Mem v0.7.17. Variants were called using GATK HaplotypeCaller v4.1.9.0 in “-ERC GVCF” mode. Any identified variant positions which were exclusive to the 518 non-reference panel accessions (i.e., those positions which were not part of the ∼35.7 M SNPs and InDels called from the reference panel) were excluded. Imputation with Beagle v5.2 was performed on the ∼35.7 M positions using the core set of 772 accessions as a reference panel to fill in any missing genotype data derived from regions of poor read coverage or quality. The effect of each of these variants was predicted using the software utility SNPEff v4.3.1t and the Ensembl GTF annotation file for the Wm82.a2.v1 reference genome.^1^ Variant annotations were restricted to the primary transcript only.

### Conversion of Variants to Alleles

The full set of ∼35.7 Mil variant positions was filtered to only include those positions predicted to cause some non-conservative amino acid change in a gene product. This includes all exonal InDels, non-sense mutations, splice site mutations, loss or gain of either a start or stop codon, and non-conservative missense mutations (as defined by groups in Supplementary Table 2). For all accessions, the remaining variant positions in each gene were concatenated to create a putative allele for all ∼55k genes in the genome. The full accession panel was then filtered to obtain only the subset of ELs for which state of origin could be determined; these 264 ELs were then used for subsequent analyses.

### Latitude Assignment and Rescaling of 264 Elite US Soybean Lines

For 141 of the 264 resequenced ELs, we were able to obtain days to maturity or relative maturity scores directly from the breeders who developed them (Supplementary Table 3). In such cases, we used those maturity scores to expand the relative latitude values of each EL to the latitudinal range encompassed by the approximate northern and southern border of their respective state. Supplementary Figure 1 illustrates this methodology using 12 resequenced ELs derived from Dr. Brian Diers’ breeding program in Illinois as an example. The northern border of Illinois has a latitude of 42.496369°N, while the southern border has a latitude of 37.231888°N. The earliest maturing EL resequenced from Dr. Diers’ program had a relative maturity (RM) value of 2.5, while the latest maturing EL was assigned an RM of 4.0. The two ELs with RM 2.5 and RM 4.0 were assigned latitude values of 42.496369°N and 37.231888°N, respectively, while the other ten ELs’ latitudes were scaled to this latitudinal range based on their RM values. ELs for which breeders provided days to maturity data, instead of RM values, were treated similarly. In such cases, ELs with the fewest number of days to maturity were assigned a latitude corresponding to the northern border of the state and ELs with the largest number of days to maturity were assigned a latitude corresponding to the southern border of the state. All ELs which matured between the earliest and latest ELs were rescaled to the latitudinal range of the state, according to their respective days to maturity score. ELs which were assigned scaled latitudes based on available maturity information are denoted by “maturity-scaled latitude” in the “LATITUDE_TYPE” column of Supplementary Table 4. ELs for which maturity scores were not available were assigned a latitude value corresponding to the centroidal latitude of the state that the EL originated from (denoted as “centroidal latitude” in the “LATITUDE_TYPE” column of Supplementary Table 4).

### *E1* and *E2* Genotype Assignment of Elite US Soybean Lines From Resequencing Data

*E1* and *E2* genotypes for all 264 ELs were determined from resequencing results. In addition to the characterized T75R substitution in the *E1* gene (Xia et al., 2012), three ELs in our resequencing panel had a frameshift mutation in *E1*, resulting from a string of adenosine repeats (Supplementary Table 5). Given that InDels which arise from sequence repeats are often artifacts of read alignment error, as well as the low frequency of this mutation among our resequencing panel, only the T75R mutation (06:20207322) was considered when assigning *e1-as/E1* genotypes to ELs. Variation at two positions in the *E2* gene was present among our resequencing panel: the previously characterized K528* non-sense mutation (10:45310798) leading to the *e2* allele (Watanabe et al., 2011), and an isoleucine to valine substitution of amino acid 220 (Supplementary Table 6). As previously stated, only non-conservative mutations were considered when differentiating alleles (as defined by amino acid groups in Supplementary Table 2). As such, *E2* genotypes were assigned solely based on the K528* non-sense mutation.

### A Curated List of Soybean Orthologs of *Arabidopsis* Flowering Time Genes Tested for Latitudinal Disparity

Wu et al. (2019) identified 420 soybean orthologs of the 215 *Arabidopsis* genes involved in flowering from a reciprocal BLASTP query. In this study, we curated an expanded list containing the 420 soybean genes identified by Wu et al., plus an additional 29 orthologs identified from a manual search of the literature (Supplementary Table 7). Genes in this list were excluded from further analysis if (1) they lacked non-conservative mutations, or (2) they lacked at least one alternate allele (i.e., non-reference allele) present in ten or more ELs. These filtering criteria resulted in a final list of 139 genes. These genes were then tested for latitudinal disparity between alleles of the subset of ELs containing the *E* genotype *e1-as/E2*. Mean comparisons were carried out using a student *t*-test (genes with two alleles) or an *ANOVA* (genes with more than two alleles). The *t*- or *f*-statistic representing the 95% confidence interval for each gene was empirically determined by randomization using 1,000 permutations. For genes with more than two alleles, significance letters were obtained from a test for least significant difference. All statistics were conducted using the “stats” package in R v4.0.2 and boxplots for genes with significant *p* values (*p* < 0.05*) were generated using ggplot2 v3.3.2.

### Weblogo Generation for Genes of Interest

Wm82.a2.v1 peptide sequences for full genes, based on the primary transcript, were pulled from Ensembl using the biomaRt v2.44.4 package. Orthologous sequences were obtained from an NCBI BLASTP query using the command line utility Protein-Protein BLAST v2.10.1. For each returned genus, the orthologous sequence with the highest percent identity was selected, while redundant sequences from each genus were discarded. Multiple sequence alignments for each gene were generated using the msa v1.20.1 R package. Weblogos were generated by clipping the multiple sequence alignment to approximately ten amino acids on either side of each variant, except in cases where variants fell within ten amino acids of the beginning or end of the aligned sequence.

## Results

### A Curated Set of Resequenced Elite US Soybean Lines

The objective for this work was to identify additional genes that may be contributing to flowering time in US soybean production environments. We performed variant calling and effect annotation on the genome sequence of 264 elite US soybean lines (ELs) from other published works (Zhou et al., 2015; Liu et al., 2020; Valliyodan et al., 2021) and from our own resequencing efforts (Supplementary Table 4). This resulted in a total of ∼11.3 Mil SNPs and InDels. Alleles were defined by the subset of SNPs and InDels which resulted in amino acid changes for each gene. This collection of ELs represents MG’s 0 through VIII and spans a latitudinal range from approximately 27.6°N to 48.9°N. A subset of 31 of these ELs have the maturity genotype *E1*/*E2*, 187 ELs have *e1-as*/*E2*, and 41 ELs have *e1-as*/*e2* (Supplementary Table 8). The 5 ELs with the maturity genotype *E1*/*e2* were excluded from further analysis (Langewisch et al., 2017).

### Soybean Orthologs of *Arabidopsis* Flowering Genes Tested for Latitudinal Disparity Between Alleles

Alleles for 139 orthologs of *Arabidopsis* flowering genes (Supplementary Table 9) were tested for significant latitudinal disparity (difference in mean latitudes based on reference or alternate allele(s) of each gene) among the 187 resequenced ELs within the *E* genotype group *e1-as/E2*. For genes that were significant (*p* < 0.05*) for disparities in mean latitude between alleles, a weblogo was created for the variants comprising each allele to determine whether each variant occurred within a conserved sequence domain which is likely important for protein function (Shaner et al., 1993). We identified a set of soybean genes likely playing a role in modulating flowering time in these ELs by taking into consideration latitudinal disparity between alleles, degree of amino acid conservation, and functional annotation of the *Arabidopsis* orthologs. Only the subset of identified genes, based on the criteria mentioned above, are discussed in further detail here; there were 19 other genes with significant latitudinal disparity that failed to meet our other criteria (Supplementary Figure 2).

### *E1* and *E2* Show Latitudinal Disparity Between Alleles for Elite US Soybean Lines

**FIGURE 2:**
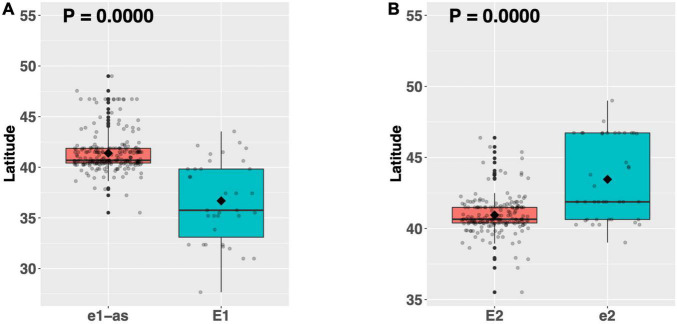
Latitudinal distribution of major alleles of **(A)** the *E1* gene and **(B)** the *E2* gene among 264 resequenced ELs. Latitude of origination was used a proxy for relative flowering time. Latitude values were estimated based on state of origin and, where available, were scaled according to maturity info provided by breeders that developed the ELs (see Experimental Procedures). Means comparisons were conducted using a student *t*-test, where the *t*-statistic representing the 95% confidence interval was empirically derived by randomization. Transparent dots represent the latitude of each accession. Boxplots show the mean (diamond), median (solid line), quartile span (box), range (vertical lines), and outliers (solid dots). The Williams 82 reference allele is on the left and the box filled with red.

Mutant alleles of *E1* (Glyma.06G207800) and *E2* (Glyma.10G221500) confer earlier flowering than their functional counterparts and are exploited to shorten the life cycle of soybean grown in northern US production environments (Watanabe et al., 2011; Xia et al., 2012; Langewisch et al., 2017). As a proof of concept, we analyzed the latitudinal disparity of alleles of *E1* and *E2* to assess whether latitude was a suitable proxy for flowering time. Among our full resequencing panel, 36 ELs had the functional allele of *E1*, while 228 ELs had the *e1-as* missense allele (Supplementary Table 8). A means comparison revealed a significant difference in mean latitude (*p* < 0.001^***^), where ELs with the *e1-as* allele had an average latitude that was 4.6°N higher than those with the *E1* allele (Figure 2A). A similar assessment for the *E2* gene was conducted using only those ELs containing the *e1-as* allele of *E1*. There were 187 ELs in our resequencing panel that had the functional *E2* allele, while 41 ELs had the non-sense *e2* allele (Supplementary Table 8). Like *E1*, alleles of *E2* showed a significant difference in mean latitude (*p* < 0.001^***^), where ELs containing *e2* were adapted to 2.5°N higher latitude, on average, than ELs containing *E2* (Figure 2B). Our findings concur with previous reports describing the allelic distribution of *E1* and *E2* among US soybean cultivars and validated our strategy of using latitude as a proxy for relative flowering time and maturity in this study (Langewisch et al., 2017). Furthermore, to avoid the large confounding effect that variation in *E1* and *E2* would have when assessing the latitudinal disparity of other genes, only the 187 ELs containing the *E* genotype *e1-as/E2* were included in further analysis.

### Modulation of Flowering Time by Members of the Autonomous Pathway

**FIGURE 3:**
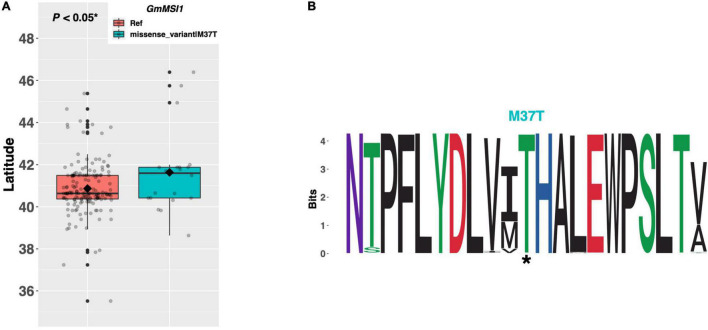
**(A)** Latitudinal distribution of *GmMSI1* alleles among 187 resequenced ELs with the maturity genotype *e1-as/E2*. Latitude of origination was used a proxy for relative flowering time. Latitude values were estimated based on state of origin and, where available, were scaled according to maturity info provided by breeders that developed the ELs (see Experimental Procedures). Means comparison was conducted using a student *t*-test, where the *t*-statistic representing the 95% confidence interval was empirically derived by randomization. Transparent dots represent the latitude of each accession. Boxplots show the mean (diamond), median (solid line), quartile span (box), range (vertical lines), and outliers (solid dots). Inset legend shows the collection of mutations which make up each allele, where “Ref” refers to the Wm82.a2.v1 reference allele. **(B)** Weblogo depicting degree of amino acid conservation of the domain surrounding each mutation (asterisk) in *GmMSI1*.

The autonomous pathway broadly modulates the plant’s ability to perceive and respond to signaling from other pathways, principally through regulation of *FLOWERING LOCUS C* (*FLC*), but also through genes that act in pathways parallel to *FLC* (Figure 1; reviewed in Simpson, 2004). *MULTICOPY SUPPRESSOR OF IRA 1* (*MSI1*), one such *FLC*-independent gene, is involved in epigenetic reprogramming of the key floral integrator gene *SUPPRESSOR OF OVEREXPRESSION OF CONSTANS 1* (*SOC1*) (Bouveret et al., 2006). *GmMSI1* on chromosome 05 (Glyma.05G131200) was identified as a putative ortholog of the *Arabidopsis MSI1* gene (Wu et al., 2019). Among our panel of resequenced ELs, 174 ELs contained the Williams 82 reference allele of *GmMSI1*, while 20 ELs contained a single methionine to threonine substitution of amino acid 37. We discovered a significant difference (*p* < 0.05*) in mean latitude between ELs with contrasting alleles of *GmMSI1*, where ELs with the M37T substitution were adapted to 0.76°N higher, on average, than ELs with the reference allele (Figure 3A). A weblogo generated from 237 genera showed the threonine residue at position 38, as well as the surrounding peptide domain, were highly conserved in this protein and thus likely important for protein function (Figure 3B).

**FIGURE 4:**
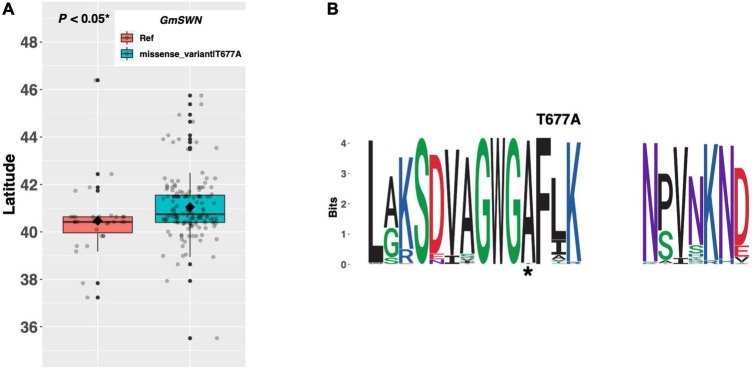
**(A)** Latitudinal distribution of *GmSWN* alleles among 187 resequenced ELs with the maturity genotype *e1-as/E2*. Latitude of origination was used a proxy for relative flowering time. Latitude values were estimated based on state of origin and, where available, were scaled according to maturity info provided by breeders that developed the ELs (see Experimental Procedures). Means comparisons were conducted using a student *t*-test, where the *t*-statistic representing the 95% confidence interval was empirically derived by randomization. Transparent dots represent the latitude of each accession. Boxplots show the mean (diamond), median (solid line), quartile span (box), range (vertical lines), and outliers (solid dots). Inset legend shows the collection of mutations which make up each allele, where “Ref” refers to the Wm82.a2.v1 reference allele. **(B)** Weblogo depicting degree of amino acid conservation of the domain surrounding each mutation (asterisks) in *GmSWN*.

*FLOWERING LOCUS C*, a potent repressor of *FT* and *SOC1*, is a key floral integrator of endogenous and external signaling from several pathways in *Arabidopsis* (Figure 1; Lee et al., 2000). Chromatin remodeling of the *FLC* locus by members of the Polycomb Repressive Complex (PRC), including the histone methyltransferase gene *SWINGER* (*SWN*), results in *FLC* silencing and derepression of *FT* and *SOC1* (Chanvivattana et al., 2004). *GmSWN* on chromosome 03 (Glyma.03G224300) was identified as a putative ortholog of the *Arabidopsis SWN* gene (Wu et al., 2019). Our resequencing panel revealed one alternate allele, in addition to the Williams 82 reference allele, containing a threonine to alanine substitution of amino acid 677. There were 37 ELs in the resequencing panel that had the reference allele, while 144 ELs had the T677A missense mutation (Figure 4A). ELs containing the alternate allele of *GmSWN* were adapted to 0.58°N higher latitude, on average, than ELs containing the reference allele (*p* < 0.05*), and a weblogo generated from 92 genera showed that the T677A substitution occurred in a region of strong conservation, where alanine was the conserved residue (Figure 4B).

**FIGURE 5:**
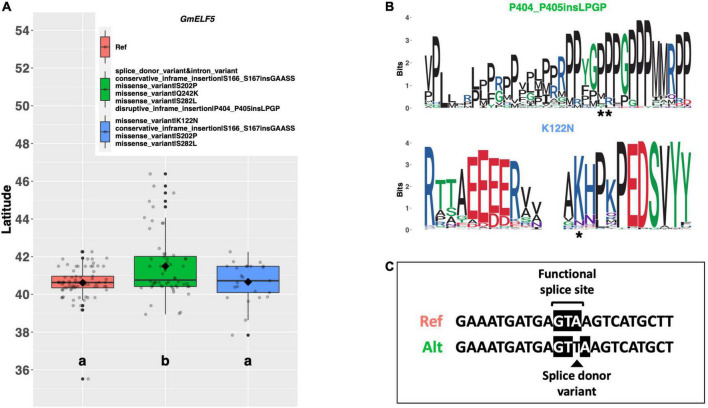
**(A)** Latitudinal distribution of *GmELF5* alleles among 187 resequenced ELs with the maturity genotype *e1-as/E2*. Latitude of origination was used a proxy for relative flowering time. Latitude values were estimated based on state of origin and, where available, were scaled according to maturity info provided by breeders that developed the ELs (see Experimental Procedures). Means comparison was conducted using an ANOVA, where the *f*-statistic representing the 95% confidence interval was empirically derived by randomization. Significance letters were obtained from a test of least significant difference. Transparent dots represent the latitude of each accession. Boxplots show the mean (diamond), median (solid line), quartile span (box), range (vertical lines), and outliers (solid dots). Inset legend shows the collection of mutations which make up each allele, where “Ref” refers to the Wm82.a2.v1 reference allele. **(B)** Weblogo depicting degree of amino acid conservation of the domain surrounding prioritized mutations (asterisk) in *GmELF5*. **(C)** Comparison of the splice site sequence at the second exon/intron boundary in the Williams 82 Ref sequence and alternate allele containing the splice donor variant.

*EARLY FLOWERING 5* (*ELF5*) is a member of the autonomous pathway in *Arabidopsis* which has been postulated to post-transcriptionally upregulate *FLC* to delay flowering (Figure 1; Noh et al., 2004). There are two alternate alleles of the soybean ortholog *GmELF5* (Glyma.05G031100) among our resequencing panel, in addition to the Williams 82 reference allele. Both alternate alleles share a glycine to lysine substitution of amino acid 242, a serine to proline substitution of amino acid 202, a serine to leucine substitution of amino acid 282, and an inframe insertion of several amino acids between amino acids 166 and 167 (Figure 5). In addition to these shared mutations, one allele has a mutated splice site and an additional inframe insertion between amino acids 404 and 405, while the other allele has a lysine to asparagine substitution of amino acid 122. A significant latitudinal disparity (*p* < 0.001^***^) was observed between the reference allele and the alternate allele containing the splice site mutation, where ELs containing the splice site mutation were adapted to 0.88°N higher latitude than ELs containing the reference allele (Figure 5A). ELs with the alternate allele with the K122N missense mutation were adapted to a similar latitude as the reference allele. A weblogo depicting each of these mutations reveals that the S202P, Q242K, S282L, and the S166_S167 insertion occur in regions of low conservation (Supplementary Figure 3). By contrast, both the P404_P405 insertion and the K122N substitution occur in apparent conserved regions, suggesting that these positions may be important for protein function (Figure 5B).

The reference allele has an intact splice site, while the allele with the splice donor variant has a single threonine insertion between the second and third nucleotide in the splice site (Figure 5C).

### Modulation of Flowering Time by Genes in the Photoperiod and Circadian Clock Pathways

**FIGURE 6:**
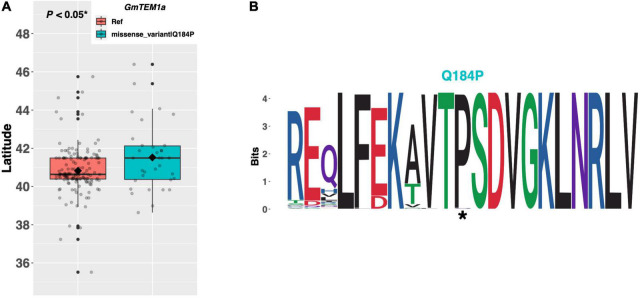
**(A)** Latitudinal distribution of *GmTEM1a* alleles among 186 resequenced ELs with the maturity genotype *e1-as/E2*. Latitude of origination was used a proxy for relative flowering time. Latitude values were estimated based on state of origin and, where available, were scaled according to maturity info provided by breeders that developed the ELs (see Experimental Procedures). Means comparison was conducted using a student *t*-test, where the *t*-statistic representing the 95% confidence interval was empirically derived by randomization. Transparent dots represent the latitude of each accession. Boxplots show the mean (diamond), median (solid line), quartile span (box), range (vertical lines), and outliers (solid dots). Inset legend shows the collection of mutations which make up each allele, where “Ref” refers to the Wm82.a2.v1 reference allele. **(B)** Weblogo depicting degree of amino acid conservation of the domain surrounding each mutation (asterisks) in *GmTEM1a*.

The *TEMPRANILLO* genes (*TEM1/TEM2*) are a family of *RAV* class transcription factors which play a complex role in mediating signaling from several pathways to delay flowering in *Arabidopsis* (Figure 1; Castillejo and Pelaz, 2008; Osnato et al., 2012). However, despite their key role in *Arabidopsis*, the effect of *TEM* orthologs on flowering time in soybean, especially *GmTEM1a* (Glyma.20G186200), is still underexplored. One alternate allele of *GmTEM1a* was discovered among the resequenced ELs in the *e1-as*/*E2* genotype group containing a glutamine to proline substitution of amino acid 184 (Figure 6A). ELs with the Q184P substitution were adapted to 0.59°N higher latitude, on average, than ELs with the reference allele (*p* < 0.05*). A weblogo generated from 124 genera revealed that the proline residue at position 184, as well as the surrounding region, is highly conserved and that the Williams 82 reference line has the non-conserved allele (Figure 6B).

**FIGURE 7:**
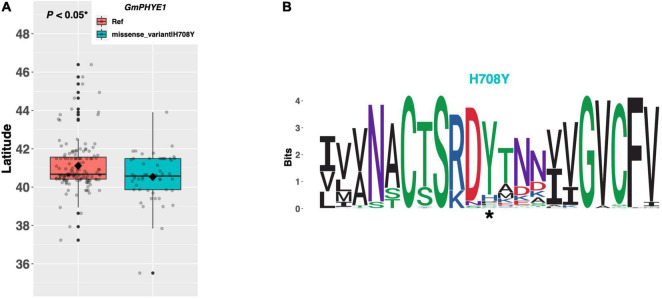
**(A)** Latitudinal distribution of *GmPHYE1* alleles among 187 resequenced ELs with the maturity genotype *e1-as/E2*. Latitude of origination was used a proxy for relative flowering time. Latitude values were estimated based on state of origin and, where available, were scaled according to maturity info provided by breeders that developed the ELs (see Experimental Procedures). Means comparison was conducted using a student *t*-test, where the *t*-statistic representing the 95% confidence interval was empirically derived by randomization. Transparent dots represent the latitude of each accession. Boxplots show the mean (diamond), median (solid line), quartile span (box), range (vertical lines), and outliers (solid dots). Inset legend shows the collection of mutations which make up each allele, where “Ref” refers to the Wm82.a2.v1 reference allele. **(B)** Weblogo depicting degree of amino acid conservation of the domain surrounding each mutation (asterisk) in *GmPHYE1*.

In *Arabidopsis*, *PHYTOCHROME E* (*PHYE*) is a photoreceptor molecule which participates broadly in plant developmental responses to light (Figure 1; Devlin et al., 1998). The soybean ortholog *GmPHYE1* on chromosome 09 (Glyma.09G088500) has a single alternate allele possessing a histidine to tyrosine substitution of amino acid 708, which is present in 53 ELs in our resequencing panel (Figure 7A). An assessment of latitudinal disparity between the Williams 82 reference allele of *GmPHYE1* and the H708Y alternate allele revealed a significant difference in mean latitudes (*p* < 0.05*), where ELs with the reference allele were adapted 0.58°N higher than ELs with the missense mutation, on average. A weblogo generated from 109 genera showed that the H708Y missense mutation occurred in a conserved protein domain, where the tyrosine residue was conserved and the histidine residue present in Williams 82 was the non-conserved residue (Figure 7B).

**FIGURE 8:**
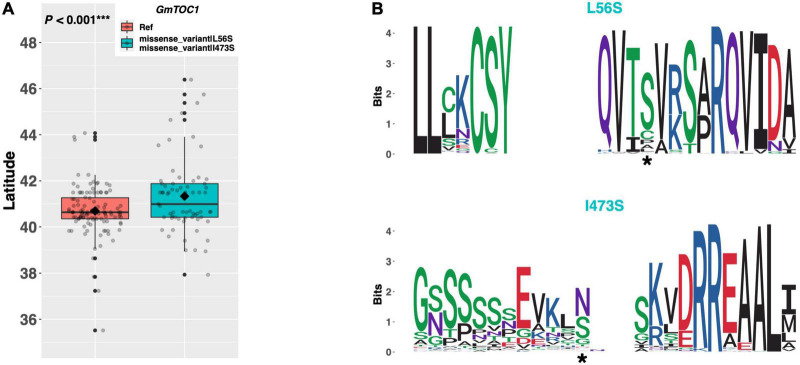
**(A)** Latitudinal distribution of *GmTOC1* alleles among 187 resequenced ELs with the maturity genotype *e1-as/E2*. Latitude of origination was used a proxy for relative flowering time. Latitude values were estimated based on state of origin and, where available, were scaled according to maturity info provided by breeders that developed the ELs (see Experimental Procedures). Means comparison was conducted using a student *t*-test, where the *t*-statistic representing the 95% confidence interval was empirically derived by randomization. Transparent dots represent the latitude of each accession. Boxplots show the mean (diamond), median (solid line), quartile span (box), range (vertical lines), and outliers (solid dots). Inset legend shows the collection of mutations which make up each allele, where “Ref” refers to the Wm82.a2.v1 reference allele. **(B)** Weblogo depicting degree of amino acid conservation of the domain surrounding each mutation (asterisk) in *GmTOC1*.

*TIMING OF CAB EXPRESSION 1* (*TOC1*) is a core member of the circadian clock complex in *Arabidopsis* and is responsible for promoting expression of genes in the evening complex (Figure 1; Gendron et al., 2012). The soybean ortholog *GmTOC1* (Glyma.06G196200) has a single alternate allele among our resequencing panel, which contains a leucine to serine substitution of amino acid 56 and an isoleucine to serine substitution of amino acid 473 (Figure 8A). An assessment of latitudinal disparity between the Williams 82 reference allele and the alternate allele revealed a significant difference in mean latitude (*p* < 0.001^***^), where ELs containing the alternate allele were adapted to 0.64°N higher than ELs with the reference allele (Figure 8A). A weblogo generated from 134 genera showed that the I473S mutation occurred in a region of low conservation, whereas the L56S mutation occurred in a region of high conservation, where the serine residue appeared to be conserved (Figure 8B).

### Modulation of Flowering Time by Genes in the Gibberellin Pathway

**FIGURE 9:**
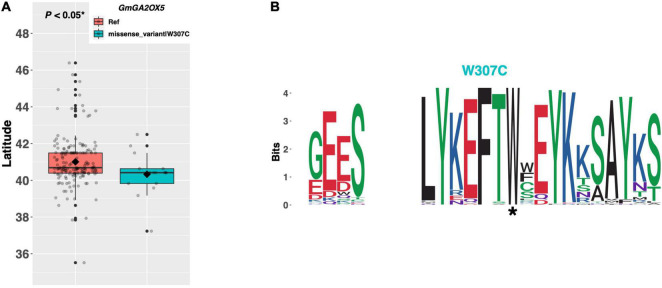
**(A)** Latitudinal distribution of *GmGA2OX5* alleles among 187 resequenced ELs with the maturity genotype *e1-as/E2*. Latitude of origination was used a proxy for relative flowering time. Latitude values were estimated based on state of origin and, where available, were scaled according to maturity info provided by breeders that developed the ELs (see Experimental Procedures). Means comparison was conducted using a student *t*-test, where the *t*-statistic representing the 95% confidence interval was empirically derived by randomization. Transparent dots represent the latitude of each accession. Boxplots show the mean (diamond), median (solid line), quartile span (box), range (vertical lines), and outliers (solid dots). Inset legend shows the collection of mutations which make up each allele, where “Ref” refers to the Wm82.a2.v1 reference allele. **(B)** Weblogo depicting degree of amino acid conservation of the domain surrounding each mutation (asterisk) in *GmGA2OX5*.

Gibberellins are a class of hormone responsible for modulating many developmental programs throughout the plant. *GmGA2OX5* and *GmGA2OX6* are orthologs of the *Arabidopsis GA2OX2* and *GA2OX1* genes, respectively, which metabolize gibberellic acid in the apical meristem and delay flowering (Figure 1). *GmGA2OX5* (Glyma.13G218200) has a single alternate allele present in 16 ELs in our resequencing panel, which contains a tryptophan to cysteine substitution of amino acid 307 (Figure 9A). We observed a significant difference in mean latitude (*p* < 0.05*) between ELs containing the Williams 82 reference allele and the alternate allele, where ELs with the reference allele were adapted to 0.67°N higher, on average, than ELs with the alternate allele (Figure 9A). A weblogo generated from 100 genera revealed that the W307C mutation occurred in a region of high conservation, where the tryptophan residue was almost perfectly conserved (Figure 9B).

**FIGURE 10:**
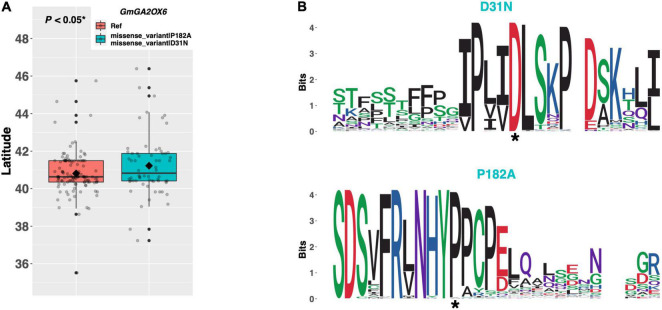
**(A)** Latitudinal distribution of *GmGA2OX6* alleles among 187 resequenced ELs with the maturity genotype *e1-as/E2*. Latitude of origination was used a proxy for relative flowering time. Latitude values were estimated based on state of origin and, where available, were scaled according to maturity info provided by breeders that developed the ELs (see Experimental Procedures). Means comparison was conducted using a student *t*-test, where the *t*-statistic representing the 95% confidence interval was empirically derived by randomization. Transparent dots represent the latitude of each accession. Boxplots show the mean (diamond), median (solid line), quartile span (box), range (vertical lines), and outliers (solid dots). Inset legend shows the collection of mutations which make up each allele, where “Ref” refers to the Wm82.a2.v1 reference allele. **(B)** Weblogo depicting degree of amino acid conservation of the domain surrounding each mutation (asterisk) in *GmGA2OX6*.

Among ELs in our resequencing panel, a single alternate allele of *GmGA2OX6* (Glyma.13G259400) existed containing an aspartate to asparagine substitution of amino acid 31, and a proline to alanine substitution of amino acid 182 (Figure 10A). An assessment of latitudinal disparity revealed a significant difference in mean latitude (*p* < 0.05*) between ELs containing the Williams 82 reference allele and ELs containing the alternate allele, where ELs with the alternate allele were adapted to 0.41°N higher latitude, on average. A weblogo generated from 102 genera revealed that both missense mutations occurred in regions of high amino acid conservation, where the aspartate and proline residues were conserved at positions 31 and 182, respectively (Figure 10B).

## Discussion

In this study, we identified an allelic series for soybean orthologs of *Arabidopsis* flowering time genes from high quality SNPs and InDels derived from resequencing analysis of the genomes of 264 elite US soybean lines (ELs). A total of 139 genes, for which allelic variation was present in high frequency among our resequencing panel, were assessed to determine the likelihood of their utilization to modulate flowering time and maturity in modern US soybean. Eight genes in various biological pathways were identified based on latitudinal disparity between alleles, amino acid conservation of identified mutations, and their function in the model organism *A. thaliana*. It should be noted that our analysis does not preclude the possibility that enrichment for these alleles in different latitudinal groups could be the consequence of breeder selection for particular alleles of linked genes, rather than selection for the genes identified in this study. However, the degree of sequence conservation, as determined by the weblogos for each variant, suggests these mutations occur in important amino acid domains of their respective proteins and thus likely have an impact on flowering time, regardless of whether they were the intended targets of selection.

### Functional Conservation and Divergence of Genes in the Autonomous Pathway

In *Arabidopsis*, *MSI1* promotes flowering *via* direct upregulation of *SOC1* and *msi1* mutants resulted in delayed flowering (Bouveret et al., 2006). Our results show that ELs with the putative functional allele of *GmMSI1* (i.e., those containing the threonine residue at position 37) are adapted to higher latitudes than ELs with the methionine residue and likely flower and mature earlier. These data suggest that the ability of *GmMSI1* to promote floral induction may be conserved in soybean and warrants further investigation as a potential target for optimizing flowering time. As is characteristic of members of chromatin remodeling complexes, mutations in *MSI1* pleiotropically affect traits other than flowering time in *Arabidopsis*, including fertilization and seed development (Köhler et al., 2003). While this seems to suggest that utilizing *GmMSI1* to optimize flowering time in soybean may result in developmental abnormalities, the fact that there is significant variation in this gene among ELs, and multiple orthologs of *MSI1* exist in soybean, suggests that this gene may have diverged substantially in function from the ancestral state.

In soybean, *GmFLC-like* acts similarly to *FLC* by physically interacting with the promoter of *GmFT2a* to inhibit its expression and delay flowering (Lyu et al., 2020). In *Arabidopsis*, the *SWN* gene is expressed in the leaf primordia and apical meristem of young seedlings, as well as in the inflorescence and floral meristems (Chanvivattana et al., 2004). By contrast, *GmSWN* transcripts were not detected in the Williams 82 reference line (Severin et al., 2010), which contains the putative non-functional allele (i.e., the allele with the threonine residue). The fact that ELs with the putative functional allele (i.e., those with the alanine residue) tend to be adapted to higher latitudes than ELs with the threonine residue, suggests that floral promotion by *GmSWN via* downregulation of *GmFLC-like* may be conserved in soybean.

*elf5* mutants in *Arabidopsis* exhibit early flowering in both long and short days at least in part by reduced promotion of the floral repressor *FLC* (Noh et al., 2004). In soybean cv. Williams 82, *GmELF5* is highly expressed in most major tissues throughout the plant (Severin et al., 2010). Our resequencing data indicated several mutations defined two distinct alternate alleles of this gene, in addition to the reference allele. Of particular interest is a mutation in a splice site region defining the second exon/intron boundary, which occurs in one of the alternate alleles. That ELs with this putative null allele are adapted to higher latitudes and likely flower earlier suggests that the function of *ELF5* to delay flowering may be conserved in soybean. The second alternate allele contained an asparagine residue in place of a conserved lysine residue at position 122; however, ELs containing this allele seem to be adapted to similar latitudes as ELs with the reference allele. This is at odds with the theory that the function of *GmELF5* to delay flowering is conserved, because we would expect ELs with the non-conserved asparagine residue to be adapted to higher latitudes. Further experimentation is needed to determine how *GmELF5* and the alleles described herein affect flowering time in soybean.

### Functional Conservation and Divergence of Genes in the Photoperiod Pathway

When functional, TEM1/TEM2 compete with the CO protein to inhibit FT expression and delay flowering in Arabidopsis (Castillejo and Pelaz, 2008). Regulatory variation in a light-responsive promoter element of the GmTEM1a paralog, GmTEM1b (also known as GmRAV), was recently shown to promote flowering time and maturity in soybean (Wang et al., 2021), confirming that the native function of GmTEM1b in delaying flowering is conserved between Arabidopsis and soybean. In contrast, ELs with the putative functional allele of GmTEM1a (i.e., those with the proline residue at position 184) are adapted to higher latitudes on average and appear to flower earlier than ELs with the glutamine residue. This finding suggests a possible divergence in function between GmTEM1a and both GmTEM1b and the Arabidopsis TEM1 gene, whereby GmTEM1a may function to promote flowering when functional. Consistent with this idea, Wu et al. showed that, among Clark near-isogenic lines containing contrasting alleles of either E1, E2, or E3, GmTEM1a was differentially expressed under long days in both the E1 and E2 NILs (Wu et al., 2019). By contrast, GmTEM1b was not differentially expressed in any of the three Clark NILs under long days. Taken together, these results suggest that GmTEM1a and GmTEM1b are regulated independently and lends credence to the theory that these genes may have divergent function.

Five phytochrome genes (*PHYA* to *PHYE*) exist in *Arabidopsis*, where *PHYA/PHYC* and *PHYB/PHYD/PHYE* form two distinct phylogenetic clades, indicating that the *PHYE* gene is most closely related to *PHYB* and *PHYD* (Mathews, 2006). In *Arabidopsis*, *phye* single mutants are identical to wild-type plants but flower early in a *phya phyb* background, revealing that the function of *PHYE* overlaps with that of *PHYA* and *PHYB* to delay flowering (Devlin et al., 1998). ELs in our resequencing panel which contained the putative functional allele of *GmPHYE1* (i.e., those with the conserved tyrosine residue) were adapted to lower latitudes, and thus likely flower later, than those containing the reference allele. *GmPHYE1* is expressed in several tissues throughout the Williams 82 reference line, including in the leaves and flowers (Severin et al., 2010). Taken together, these results suggest that the function of *PHYE* to delay flowering is likely conserved in the soybean ortholog *GmPHYE1*.

The *Arabidopsis TOC1* gene is a core component of the circadian clock involved in reciprocal regulation of genes that stimulate the morning complex (*CCA1* and *LHY*), and impairment of which results in a shortened circadian period and concomitant early flowering (Strayer et al., 2000; Gendron et al., 2012). A novel allele of *GmTOC1* (Glyma.06G196200) containing a conserved serine residue at position 56 was identified among ELs in our resequencing panel, and ELs containing this allele were adapted to higher latitudes than ELs with the leucine residue at this position. Liu H. et al. (2009) identified a conserved pseudo-receiver domain near the N terminus of the *GmTOC1* paralog on chromosome 04 (Glyma.04G166300) and demonstrated that its expression peaks in the evening, similar to that of *TOC1* in *Arabidopsis*. These data suggest that the function of *TOC1* to maintain appropriate circadian period, and thus to delay flowering, appears to be conserved in at least the *GmTOC1* gene on chromosome 04. By contrast, those ELs in our resequencing panel with the putative functional allele of the *GmTOC1* gene on chromosome 06 (i.e., those with the serine residue) appear to flower earlier, suggesting that there may be divergence in function between *GmTOC1* on chromosome 06 and the *Arabidopsis TOC1* gene.

### Functional Conservation and Divergence of Genes in the Gibberellin Pathway

Biosynthesis of florigenic gibberellic acid (GA) is mediated by *GA20OX2* in the leaves of *Arabidopsis*, the loss of function of which delays flowering modestly under long days and dramatically under short days (Rieu et al., 2008b). By contrast, the *GA2OX* class of oxidases inactivate GA, and mutants of which result in earlier flowering, especially in short days (Rieu et al., 2008a). However, reports describing the activity of GA reveal that the effects and precise mechanism by which GA influences photoperiod-dependent flowering have significantly diverged between species (reviewed in Mutasa-Göttgens and Hedden (2009)). Our results revealed a single alternate allele in each of two *GA2OX* orthologs, *GmGA2OX5* and *GmGA2OX6*. ELs in our resequencing panel containing the putative functional allele of *GmGA2OX5* (i.e., those with the tryptophan residue at position 307) are adapted to higher latitudes and likely flower earlier than those with the cysteine residue, suggesting that *GmGA2OX5* may have diverged in function from its *Arabidopsis* ortholog, *GA2OX*. By contrast, ELs containing the putative functional allele of *GmGA2OX6* (i.e., those with the aspartate residue at position 31 and the proline residue at position 182) were adapted to lower latitudes than ELs with the putative impaired allele, suggesting that *GmGA2OX6* may delay flowering, similar to its *Arabidopsis* ortholog *GA2OX*. However, further work is needed to test how each of these genes and the alleles described here affect flowering time in cultivated soybean.

### Future Research

The candidate genes identified from this exploratory analysis constitute the preliminary framework needed to further define the genetic architecture of flowering time in US soybean. Given that the latitudinal separation between alleles of these genes is generally smaller than that of *E1* and *E2*, it’s likely that these candidates are minor effect genes, however, further work is needed to validate these as modulators of flowering time and to characterize the magnitude of their effects under natural photoperiods. The fact that eight candidate genes were identified means there are a multitude of allele combinations that may be present in any one accession. When investigating the allele status for the combination those eight genes, accessions with a larger number of the “northern” alleles (the allele with the higher mean latitude) were generally adapted to more northern latitudes, and those with fewer “northern” alleles were adapted to more southern latitudes. Three accessions had the “northern” alleles of all eight candidate genes (PI548540, SS202, and Amcor89), while five accession had just one “northern” allele (PI597382, PI548387, PI548565, Avery, and SA17-15682). Without further research to understand the impact of each identified candidate gene individually, it is difficult to model what is likely to be complex interactions of these genes in relation to photoperiod response. Recent work established a regulatory link between the *GmTEM1a* ortholog, *GmTEM1b* (*GmRAV*), and several gibberellins-associated genes in the modulation of plant height in soybean (Xue et al., 2022). It would be interesting to investigate whether there is a similar link between the *GmTEM1a*, *GmGA2OX5*, and *GmGA2OX6* genes identified in this study in the control of flowering.

## Data Availability Statement

The datasets presented in this study can be found in online repositories. The names of the repository/repositories and accession number(s) can be found below: https://soykb.org/public_data.php, KB100.

## Author Contributions

ND and KB conceived and designed experiments, analyzed data, and wrote the manuscript. YC and TJ developed the resequencing analysis pipelines, generated the combined sequence data files, and provided data quality control. AS, GG, BD, AL, DW, DH, and MH provided germplasm and phenotypes for the analysis. All authors have read and approved the manuscript.

## Conflict of Interest

The authors declare that the research was conducted in the absence of any commercial or financial relationships that could be construed as a potential conflict of interest.

## Publisher’s Note

All claims expressed in this article are solely those of the authors and do not necessarily represent those of their affiliated organizations, or those of the publisher, the editors and the reviewers. Any product that may be evaluated in this article, or claim that may be made by its manufacturer, is not guaranteed or endorsed by the publisher.

## Footnotes

1
http://ftp.ebi.ac.uk/ensemblgenomes/pub/release-51/plants/gtf/glycine_max

## Supplementary Material

Supplementary Figure 1 Example of latitudinal rescaling according to days to maturity or relative maturity scores. Skim resequenced US cultivars (dots) derived from Dr. Brian Diers’ breeding program. Latitude values **(left)** were scaled according to their Relative Maturity values **(right)** to fit the approximate latitudinal range of the state of Illinois.

Supplementary Figure 2 Boxplots of genes with a significant latitudinal disparity between alleles from among 187 resequenced ELs with the maturity genotype *e1-as/E2*. Latitude of origination was used a proxy for relative flowering time. Latitude values were estimated based on state of origin and, where available, were scaled according to maturity info provided by breeders that developed the cultivars (see Experimental Procedures). Means comparison was conducted using an *ANOVA*, where the *t*-statistic representing the 95% confidence interval was empirically derived by randomization. *P* value refers to significance between alleles with the largest difference in means. Transparent dots represent the latitude of each accession. Boxplots show the mean (diamond), median (solid line), quartile span (box), range (vertical lines), and outliers (solid dots). Inset legend shows the collection of mutations which make up each allele.

Supplementary Figure 3 Weblogo depicting degree of amino acid conservation of the domain surrounding each mutation (asterisk) in *GmELF5*.
