# Supplementary figures and images for: Candidate Genes Modulating Reproductive Timing in Elite US Soybean Lines Identified in Soybean Alleles of Arabidopsis Flowering Orthologs With Divergent Latitude Distribution
Source: Front Plant Sci. 2022 Apr 29;13:889066. doi: 10.3389/fpls.2022.889066 (PMC9100572; doi:10.3389/fpls.2022.889066)

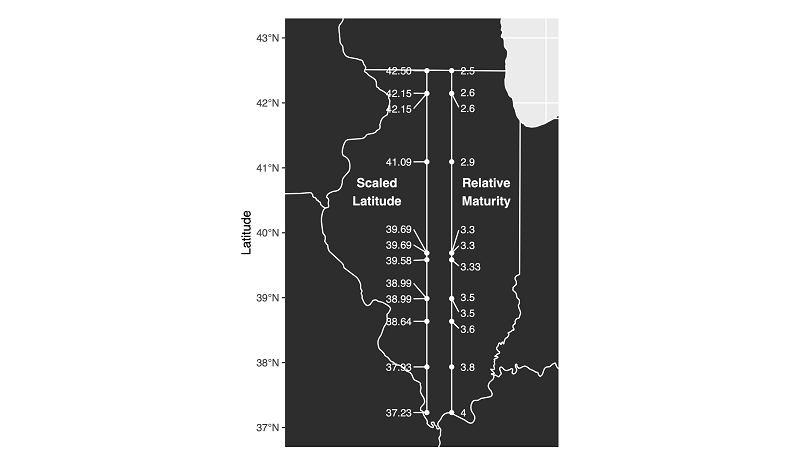

Supplement: Supplementary Figure 1 — Example of latitudinal rescaling according to days to maturity or relative maturity scores. Skim resequenced US cultivars (dots) derived from Dr. Brian Diers’ breeding program. Latitude values (left) were scaled according to their Relative Maturity values (right) to fit the approximate latitudinal range of the state of Illinois. [file Image_1.TIF]

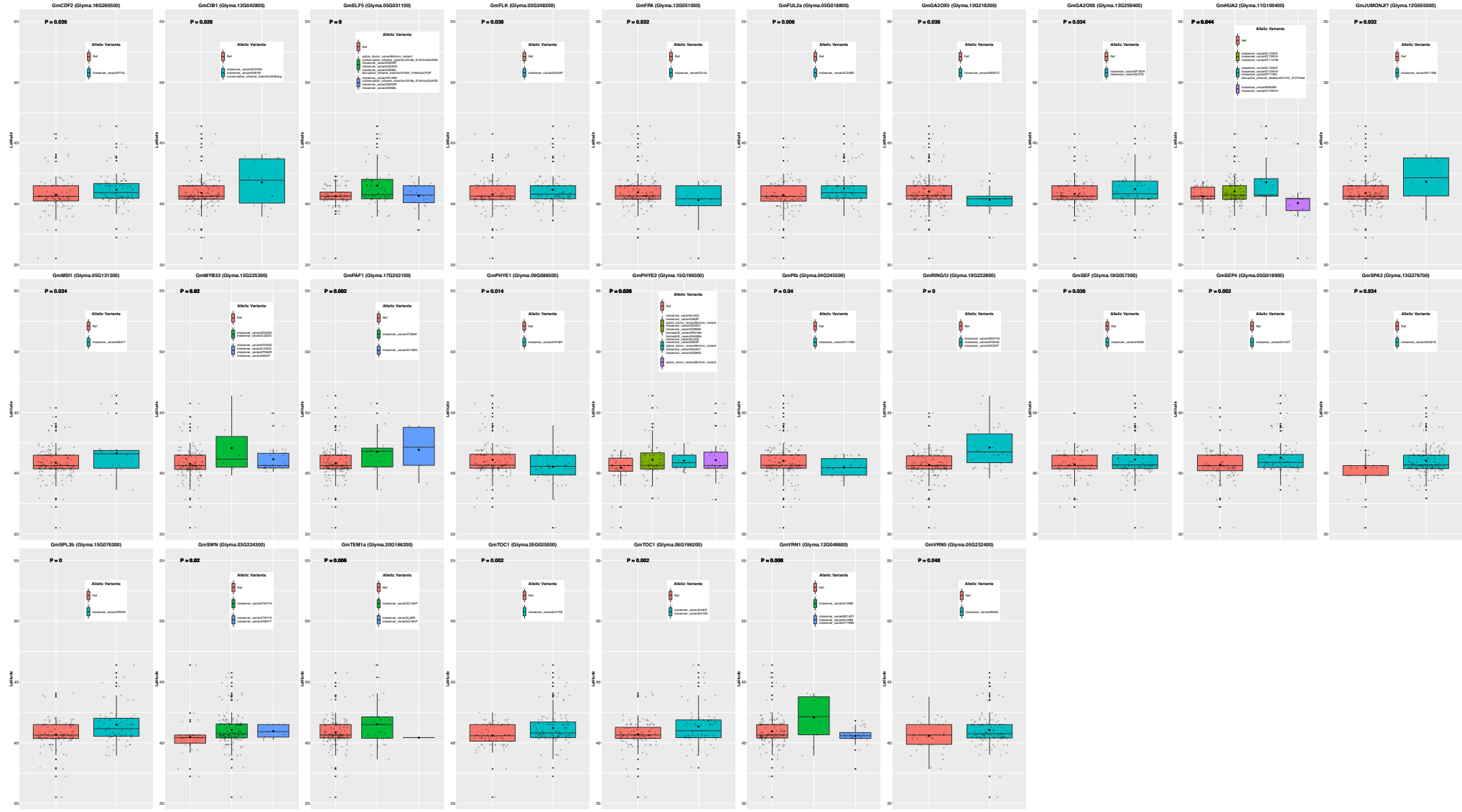

Supplement: Supplementary Figure 2 — Boxplots of genes with a significant latitudinal disparity between alleles from among 187 resequenced ELs with the maturity genotype e1-as/E2. Latitude of origination was used a proxy for relative flowering time. Latitude values were estimated based on state of origin and, where available, were scaled according to maturity info provided by breeders that developed the cultivars (see Experimental Procedures). Means comparison was conducted using an ANOVA, where the t-statistic representing the 95% confidence interval was empirically derived by randomization. P value refers to significance between alleles with the largest difference in means. Transparent dots represent the latitude of each accession. Boxplots show the mean (diamond), median (solid line), quartile span (box), range (vertical lines), and outliers (solid dots). Inset legend shows the collection of mutations which make up each allele. [file Image_2.pdf]

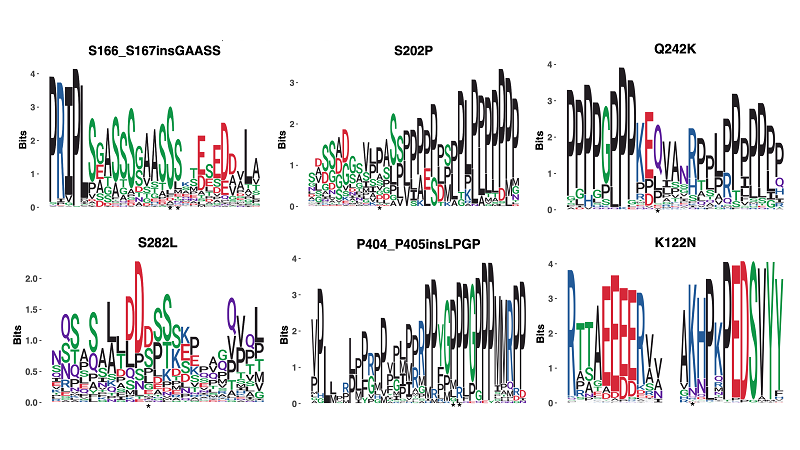

Supplement: Supplementary Figure 3 — Weblogo depicting degree of amino acid conservation of the domain surrounding each mutation (asterisk) in GmELF5. [file Image_3.TIF]
